# Design of a novel tri-axis ZnO nanowires based piezoelectric accelerometer

**DOI:** 10.1371/journal.pone.0318069

**Published:** 2025-03-05

**Authors:** Muhammad Sohaib Khan, Hassan Elahi, Muhammad Mubasher Saleem, Masood Ur Rehman, Muhammad Abdullah Tayyab, Mohsin Islam Tiwana

**Affiliations:** 1 Department of Mechatronics Engineering, National University of Sciences and Technology, Islamabad, Pakistan

## Abstract

Micro-Electromechanical Systems (MEMS) are pivotal in modern technology, serving as components like accelerometers, gyroscopes, and pressure sensors in various applications. MEMS accelerometers are key components used for measuring motion and vibrations in a wide range of systems. This paper presents the proposed design of a ZnO nanowires-based piezoelectric accelerometer. Owing to the ZnO nanowires’ unique piezoelectric properties, the accelerometer can measure acceleration in three axes. A mathematical model is derived to analyze the behavior of nanowires under applied acceleration. Finite Element Method (FEM) simulations were carried out to evaluate the performance of the accelerometer. The key parameters of the accelerometer such as mechanical deformation, stress, voltage, and sensitivity are evaluated while applying a dynamic acceleration of 0.1 g and static acceleration of up to 50 g. The simulation results show a sensitivity of 0.25 V/g for an applied acceleration in the x and y axes (in-plane acceleration) and 1.40 V/g sensitivity was achieved in the z-axis (out-of-plane acceleration). The acceleration analysis reveals that the range and sensitivity of the sensor are high, that it can measure acceleration in three axes, and it also shows a linear behavior under static acceleration. The proposed accelerometer’s tri-axis acceleration sensing and self-powered capability make it an excellent choice for integration in biomedical applications.

## Introduction

Micro-Electromechanical Systems (MEMS) have played an important role in the miniaturization of sensors and actuators across a wide range of industries, including automotive safety systems, aerodynamics, haptics, consumer electronics, and biomedical devices [1–4]. MEMS devices offer a unique and vast array of functionalities, by miniaturizing mechanical, electrical, and signal processing components onto a single chip. MEMS devices can serve as sensors such as accelerometers, gyroscopes, tactile sensors, and pressure sensors [5–8] or actuators like microswitches, micromirrors, and micropumps [9–11]. MEMS devices have become essential in modern technology due to their ability to function as highly efficient sensors and actuators. MEMS accelerometers are crucial components that convert acceleration into an electrical signal. These devices are an integral part of modern technology, being used in smartphones to detect screen orientation, bridges and buildings for structural health monitoring, in cars for airbag deployment systems, and in wearable fitness devices to monitor physical activity [12–14]. The compact size, low power consumption, high sensitivity, and cost-effectiveness of MEMS accelerometers make them ideal for integration into various applications, ranging from consumer electronics to critical safety systems.

In the literature, different transduction mechanisms reported, including capacitive, piezoresistive, inductive, optical, and piezoelectric that have been used to develop accelerometers [12, 15–18]. Each transduction mechanism has some pros and cons. The capacitive-based accelerometers are mostly used as they offer high sensitivity, but their main disadvantage is that they have complex read-out circuitry and suffer from nonlinearity. Piezoresistive accelerometers are used because of their simple construction and low cost but they consume high power and have hysteresis issues. In inductive accelerometers, the output is linear along with their high dynamic range, but the main problem is their low-frequency response and low reliability. Optical accelerometers give an advantage of high resolution but are very costly and complex in structure. Accelerometers based on the piezoelectric transduction mechanism offer more advantages including high sensitivity, frequency response, accuracy, and dynamic range that are often superior to other transduction mechanisms.

Various piezoelectric materials such as PZT, AlN, BaTiO_3_, GaN, PVDF, and ZnO are reported in the literature [3, 12, 19–22]. Lead-based materials have high piezoelectric coefficients, but their toxicity limits their application, especially in consumer and biomedical devices. Other materials may have lower piezoelectric coefficients, but they are often chosen for their environment friendliness and biocompatibility. ZnO as a piezoelectric material offers several advantages over other materials due to their higher piezoelectric coefficients, ease of fabricating, and biocompatibility [3, 23–25]. The use of ZnO in the form of nanowires offers several advantages over thin films because of its unique one-dimensional nanostructure. Due to their high surface-to-volume ratio, nanowires enhance sensitivity and enable more efficient stress transduction [25, 26]. Additionally, ZnO nanowires offer more mechanical flexibility compared to ZnO thin films, making them ideal for applications that require dynamic responsiveness and durability. The nanowires reported in the literature are circular [3, 20, 27] as well as square in shape [25, 28, 29]. Each shape has some pros and cons. Square-shaped nanowires are preferred over circular nanowires because of their flexural rigidity, more uniform deformation along the axis, and also due to their higher piezoelectric efficiency as they generate higher piezoelectric potential at their edges as compared to circular nanowires. Utilizing ZnO nanowires in a multi-axis accelerometer configuration leverages these properties to achieve high sensitivity and resolution across all three dimensions. The combination of MEMS technology with nanostructured materials like ZnO nanowires represents a significant advancement in the development of accelerometers, offering enhanced performance while maintaining compactness and cost-effectiveness.

Kim et al. developed an accelerometer based on ZnO nanowire-based piezoelectric material that measured uniaxial acceleration of up to 1 g in the 50–500 Hz frequency range. The authors used Silver Epoxy as a top and bottom electrode, which was a limitation of the accelerometer as the silver epoxy that was bonded with the sensor needed to be a uniform layer which was difficult to attain [3]. Koka et al. fabricated an accelerometer using BaTiO_3_ nanowires as a piezoelectric material to measure the acceleration in normal axis up to 1 g in the 100–1000 Hz frequency range. The main limitation of the design is that BaTiO_3_ requires high voltage poling of 75 kVcm^−1^ to ensure that the dipoles in the nanowires arrays align in the electric field direction [20]. Wang et al. developed a ZnO nanowires paper-based accelerometer that can measure acceleration in one direction in the frequency range of up to 200Hz. The main issue with this design was that its sensitivity was as low as 16 mV/g [18]. Ramany et al. fabricated a vanadium-doped ZnO nanowire accelerometer and compared the output voltage and sensitivity of a vanadium-doped ZnO nanowire accelerometer with an undoped ZnO nanowire accelerometer. The sensitivity is increased from 1.71 V/g to 1.93 V/g [30]. Another accelerometer was developed by Ramany et al. in which they fabricated a Nickel Vanadium doped ZnO nanowire accelerometer. The authors reported a sensitivity of 6.9 V/g which was very high [31]. However, the main issue with these nanowires-based accelerometers was that they can only measure acceleration along a single axis. To the best of our knowledge, no prior studies have reported the development of tri-axis accelerometers in the literature.

This research paper focuses on the design and analysis of a novel multi-axis piezoelectric MEMS accelerometer by exploiting the unique properties of ZnO nanowires. The main aim is to measure acceleration in the tri-axis and enhance their sensitivity, and range. The proposed design is also compatible with MEMS fabrication techniques.

## Design of piezoelectric sensor

**Fig 1:**
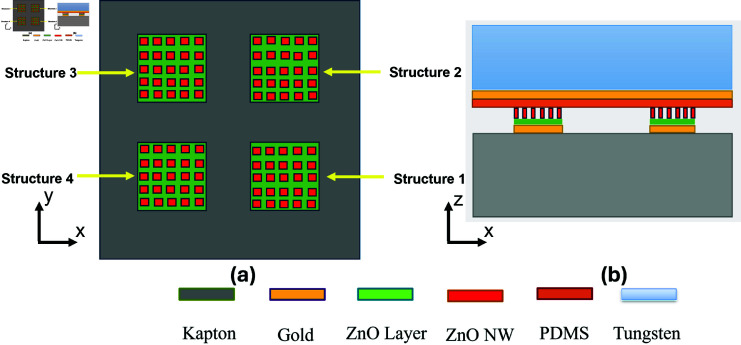
Schematic of proposed accelerometer (a) Top view (b) Side view.

**Fig 2:**
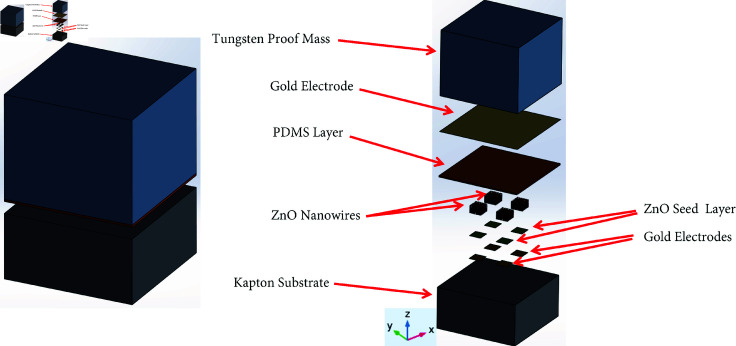
Design of proposed accelerometer (left) Isometric view (right) Exploded view.

The sensor design is proposed by utilizing the piezoelectric properties of ZnO nanowires. The bottom layer/substrate of the proposed accelerometer is Kapton. The Kapton substrate is preferred over Silicon because of its flexible behavior [28, 29, 32]. Next, four gold electrodes are patterned on the substrate and these gold electrodes act as a voltage terminal. Then, the ZnO seed layer is deposited on the top of gold electrodes, so that the ZnO nanowires can be grown easily. On the surface of the ZnO seed layer, the piezoelectric ZnO nanowires are grown such that they are only grown on the seed layer. Next, a dielectric layer is deposited over the top of ZnO nanowires to increase the accelerometer’s sensitivity. PDMS has been chosen as a dielectric layer material because of its high dielectric constant and it also acts as an adhesive layer used to stick the top electrode. On the top of the PDMS layer, a thin layer of gold is patterned on it, which acts as a top electrode. Lastly, a proof mass is placed on the top of the entire structure. Tungsten has been used as a proof mass because of its high density. The schematics of the proposed accelerometer are shown in Fig 1. Similarly, the 3d model, and exploded view of the proposed accelerometer are shown in Fig 2.

## Working principle

ZnO nanowires exhibit unique piezoelectric property, making them suitable for different sensing applications. Specifically, ZnO nanowires generate a negative voltage when they experience compressive force and a positive voltage when subjected to tensile force. Due to this unique property of generating positive and negative voltages, ZnO nanowires can effectively measure acceleration in multiple axes. So, we have grown four unique structures of nanowires to utilize this exceptional property to measure acceleration in the tri-axis. In the accelerometer, when acceleration is applied along the normal direction (z-axis), all four structures of ZnO nanowires are compressed. Due to the piezoelectric properties of ZnO nanowires, all four structures generate the same voltage. When an acceleration is applied in the x-direction, structures 1 and 2 experience compressive stress while structures 3 and 4 experience tensile stress. Due to this compressive and tensile stress, the ZnO nanowires structures produce opposite voltages. Similarly, when the input acceleration is applied in the y-direction, structures 1 and 4 experience tensile stress but structures 2 and 3 experience compressive stress. Due to this compressive and tensile stress behavior, the voltages produced by the ZnO structures are in opposite directions [28, 33].

## Mathematical model

An analytical model has been developed to describe the behavior of the proposed accelerometer to complement the FEM simulations. This mathematical model helps us to understand the piezoelectric response of ZnO nanowires and the resulting voltage under applied acceleration. The applied acceleration is firstly converted into force by calculating its mass by using Eq 1


$$\begin{array}{c} m=\rho V \end{array}$$ (1)

Here m is the mass, *ρ* is the density, and V is the volume of the proof mass.

In Eq 1 the mass is calculated by considering the density and volume of tungsten. By using Newton’s second law, the acceleration is converted into force. This force is crucial for evaluating the mechanical deformation of the ZnO nanowires. Now, consider a single nanowire acting as a cantilever beam, with one end fixed while the other is subjected to the load. As a result, deformation occurs along the length of the nanowire. The moment of inertia of the single nanowire can be determined using [34].


$$\begin{array}{c} I=\frac{bh^{3}}{12} \end{array}$$ (2)

Here I is the moment of Inertia, b is the width of the nanowire, and h is the height of the nanowire.

The moment of Inertia is the measure of the nanowire’s resistance to bending under the applied force, which directly affects the stress and strain. Next, the spring constant is calculated to determine the stiffness of the ZnO nanowires. The spring constant is calculated by using


$$\begin{array}{c} k=\frac{3EI}{h^{3}} \end{array}$$ (3)

Here k is the spring constant, and E is Young’s modulus of the ZnO nanowires.

The spring constant directly affects the displacement of the nanowire. The displacement of the nanowire is calculated by using Hooke’s law. This displacement is important in determining the strain, directly affecting the generated charge and voltage. Another important factor that affects the piezoelectric response is the bending moment of the nanowire. The bending moment is calculated by using


$$\begin{array}{c} M=Fh \end{array}$$ (4)

In M is the bending moment of the nanowire.

The bending moment results in axial stress on the nanowires, with maximum stress occurring at the nanowire’s fixed end. The stress on the nanowire can be calculated by using [34, 35]


$$\begin{array}{c} \sigma =\frac{Mc}{I}=\frac{Mh}{2I} \end{array}$$ (5)

where $c=\frac{h}{2}$ is the distance from the neutral axis, and *σ* is the stress.

Stress is the critical parameter that can influence the piezoelectric voltage of the nanowire. After finding the stress, the electrical displacement can be calculated by using [35]


$$\begin{array}{c} D=d_{33}\sigma \end{array}$$ (6)

Here D is the electrical displacement and *d*_33_ is the piezoelectric constant.

The electric displacement is the amount of electric charge generated per unit area. The electrical field is also an important factor in calculating the generated voltage. The electrical field is directly proportional to the electrical displacement, calculated by using Eq 7


$$\begin{array}{c} E=\frac{D}{\epsilon_{0}\epsilon_{pdms}} \end{array}$$ (7)

Here E is the electrical field, $\epsilon_{0}$ is the absolute permittivity, and $\epsilon_{pdms}$ is the permittivity of PDMS.

The electric field is a measure of the voltage generated across the nanowires and $\epsilon_{pdms}$ is considered due to the presence of a PDMS layer between the ZnO nanowires and the top electrode. The generated voltage can be calculated by using Eq 8


$$\begin{array}{c} V=Eh \end{array}$$ (8)

Here V is the generated voltage.

The analytical model highlights the stress distribution, displacement, and generated electrical output, demonstrating the sensor’s capability. By integrating this analytical model with FEM simulations, it offers useful insights into the performance of the sensors and validates its generated voltage for advanced sensing applications.

**Fig 3:**
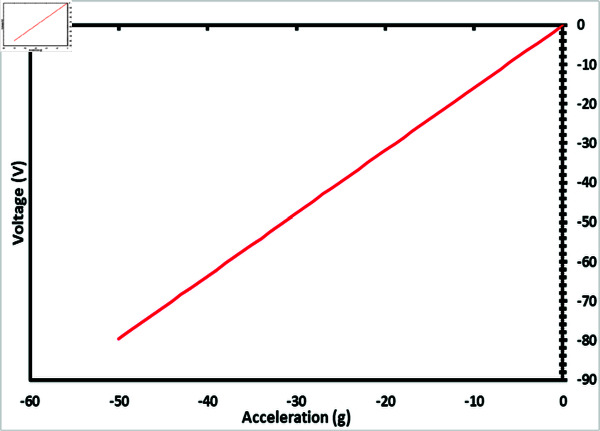
Calculated generated voltage vs applied acceleration by using mathematical modelling.

To validate the performance of the accelerometer, a detailed analysis of the generated voltage in the z-axis when the acceleration is applied along the z-axis. The acceleration is applied in the downward direction i.e. negative z-axis. The model predicts the generated voltage and the sensitivity of the proposed accelerometer which is 1.59 V/g when static acceleration is applied up to 50 g. Fig 3 illustrates the calculated value of generated voltage as a function of applied acceleration. Due to the compression of nanowires, the generated voltage is negative, therefore in the mathematical modeling, the generated voltage is considered negative.

## FEM simulation

**Fig 4:**
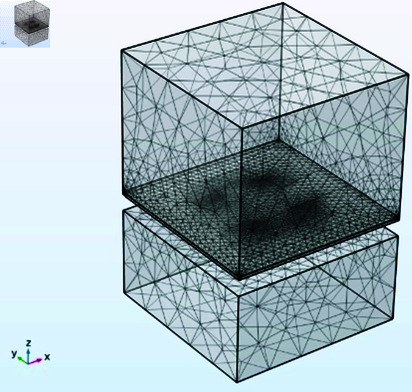
Meshed model of proposed accelerometer.

FEM simulations are performed to evaluate the performance of the ZnO nanowires for multi-axis acceleration measurement. The main objective of performing FEM simulation is to analyze the mechanical and electrical response of the accelerometer under static and dynamic acceleration. The proposed design of a piezoelectric accelerometer is numerically simulated by utilizing COMSOL Multiphysics software. The simulations provided useful insights into the relationship between applied acceleration and the stress distribution, deformation, and generated voltage. Various study modes are used to simulate the behavior of the accelerometer. An Eigenfrequency study is used to find out the Mode shapes of the accelerometer. Mode shapes are used to determine the behavior of the accelerometer in an undamped, unforced environment. Frequency Domain study is used to simulate the behavior of the accelerometer under an action of dynamic acceleration. Similarly, the Stationary study is used to find out the behavior of the accelerometer when static acceleration is applied. A 3d image of the meshed model of the accelerometer is shown in Fig 4.

The proposed sensor consists of 290 x 290 *μ*m of Kapton substrate having a thickness of 200 *μ*m. Four layers of 77.5 x 77.5 *μ*m gold, are patterned on the top of Kapton for the bottom electrode with a thickness of 0.5 *μ*m and the distance between them is 45 *μ*m. Next, four ZnO seed layers are stacked on top of the Gold Electrode which has the same dimensions as of gold electrode, but their thickness is 0.1 *μ*m. After that, a 15 x 15 array of ZnO nanowires is created on each seed layer, which means four arrays of ZnO nanowires are created. The height of each nanowire is 40 *μ*m, while the length and width of each nanowire is 2.5 *μ*m and the spacing between them is also 2.5 *μ*m. The distance between the nanowires is taken as 2.5 *μ*m to allow their sufficient freedom of movement as reported by Wood et al. [29]. A layer of PDMS of 8 *μ*m is placed on top of nanowires having the size of 290 x 290 *μ*m. On top of the PDMS layer, a thin gold layer with a thickness of 0.5 *μ*m is patterned, acting as the top electrode with the same area as the PDMS. Then a proof mass of tungsten is placed on the top of gold electrodes having dimensions of 290 x 290 x 290 *μ*m. Dynamic and Static accelerations are applied to the accelerometer to test the proposed design. A dynamic acceleration of 0.1 g is applied at various frequencies along all three axes, and a static acceleration of up to 50 g is also applied along all three axes. The stress, deformation, and generated voltage of the ZnO nanowires are analyzed for the applied acceleration.

## Mode shapes

**Fig 5:**
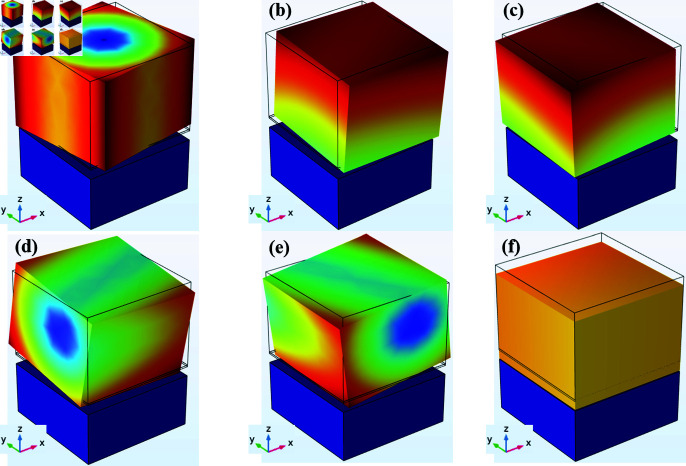
Mode shapes of the proposed accelerometer (a) 1073 Hz (b) 1426 Hz (c) 1433 Hz (d) 3883 Hz (e) 3902 Hz (f) 6647 Hz.

Mode shapes are critical in understanding the behavior of piezoelectric accelerometers. Mode shapes tell us how the structure deforms at certain frequencies, which plays an important role in stress distribution and voltage generation in nanowires. Among the six mode shapes analyzed, only the 4th, 5th, and 6th are considered, while the first three are excluded. Fig 5(a) shows the 1st mode shape of the accelerometer where the movement of the accelerometer is characterized by twisting. Fig 5(b) and 5(c) illustrate the 2nd and 3rd mode shapes of the accelerometer, where the movement of the accelerometer is diagonally flexure. The first three mode shapes occurred at a lower frequency, but they are excluded because of their direction of deformation. Due to the accelerometer’s twisting and diagonally flexure movement, the distribution of stress on the nanowires is uneven and the magnitude of stress is insufficient, which affects the voltage generation. Due to these reasons, the 4th, 5th, and 6th mode shapes are considered because of their ability to produce maximum and uniform stress and generate the maximum voltage on the nanowires. The 4th mode shape which occurs at 3883 Hz is shown in Fig 5(d).

It exhibits shear deformation along the y-axis. This mode shape is mainly used to measure the acceleration in the y-axis because at this frequency the structures deforms such that structures 1 and 4 experience tensile stress while the other two which are 2 and 3 experience compressive stress. This unique behavior at this mode shape makes it ideal for Y-axis measurement. At 3902 Hz, the 5th mode shape shown in Fig 5(e) occurs. It exhibits shear deformation along the x-axis. This mode shape is used to measure the acceleration in the x-axis because of a similar behavior to the 4th mode shape. The only difference is that structures 1 and 2 experience compressive stress but structures 3 and 4 experience tensile stress. Due to this behavior, the 5th mode shape is optimal for X-axis acceleration measurements. Similarly, the 6th mode shape, shown in Fig 5(f), occurs at 6647 Hz and exhibits normal deformation along the z-axis. This mode shape helps measure acceleration in the z-axis because of the movement of the sensor at this frequency. When the sensor oscillates all four structures are deformed and produce identical stress and voltage. Due to this identical voltage at all structures, this frequency is ideal for Z-axis acceleration measurement.

## Results and discussion

**Fig 6:**
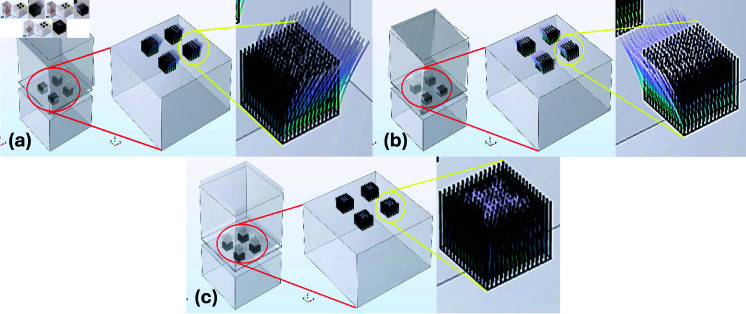
Stress distribution on ZnO nanowires when an acceleration of 0.1 g is applied in the direction of (a) X-Axis (b) Y-Axis (c) Z-Axis

**Fig 7:**
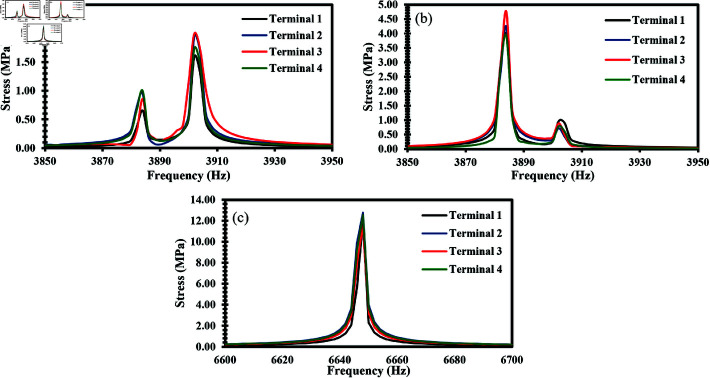
Stress vs frequency response graph of ZnO nanowires when an acceleration of 0.1 g is applied in the direction of (a) X-Axis (b) Y-Axis (c) Z-Axis.

A dynamic acceleration test was performed with an input of 0.1 g across all three axes to determine the stress output of the proposed sensor. As the desired modal frequencies in all axes were different, the performance of the accelerometer was analyzed at various frequency ranges. The effect of stress on ZnO nanowires subjected to acceleration was analyzed as it is an important factor in determining the generated voltage. Fig 6(a), 6(b), and 6(c) present the FEM simulation as a stress output when an acceleration was applied along the x-axis, y-axis, and z-axis respectively. Fig 7(a) shows the response of frequency vs stress when acceleration was applied along the x-axis. The maximum stress occurred at 3902 Hz at its modal frequency. Similarly, Fig 7(b) presents the output stress vs frequency when acceleration was applied along the y-axis. The maximum stress was observed at 3882 Hz which is also the modal frequency of the accelerometer in the y-axis. Fig 7(c) illustrates the frequency and stress relationship when acceleration was applied along the z-axis. The peak stress occurred at 6648 Hz.

**Fig 8:**
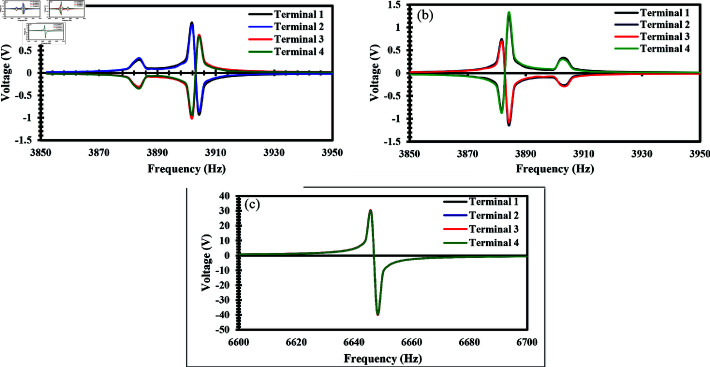
Frequency vs generated voltage graph of ZnO nanowires when an acceleration of 0.1 g is applied in the direction of (a) X-Axis (b) Y-Axis (c) Z-Axis.

In the simulation, a dynamic acceleration of 0.1 g was applied to the proof mass of the accelerometer in all three axes to determine the generated voltage on ZnO nanowires. The tri-axis accelerometer was analyzed at various frequencies as the modal frequency in each axis is different. When an acceleration of 0.1 g is applied along the x-axis, the performance of the accelerometer was analyzed in the frequency range of 3850-3950 Hz having a step of 2 Hz. Structures 1 and 2 experience compressive stress while structures 3 and 4 experience tensile stress. Due to the compressive and tensile behavior of the nanowires, structures 1 and 2 experience the same voltage, while structures 3 and 4 generate the same voltage but with opposite polarity relative to structures 1 and 2 [28]. The high output voltage is reported at 3902 Hz. Fig 8(a) shows the results between the frequency and voltage when the accelerometer experiences an acceleration of 0.1 g in the x-axis. Similarly, an acceleration of 0.1 g was applied along the y-axis and the operating range of frequency was 3850–3950 Hz with a step of 2 Hz. Due to the applied acceleration, structures 1 and 4 experience compressive stress while structures 2 and 3 experience tensile stress. As a result, structures 1 and 4 generate the same voltage, and structures 2 and 3 generate the same voltage, but structures 2 and 3 experience opposite polarity of voltage compared to structures 1 and 4 [28].

The highest voltage generated by the nanowires was observed at 3882 Hz. Fig 8(b) presents the frequency vs voltage graph when an acceleration of 0.1 g is applied along the y-axis. Similarly, when an acceleration of 0.1 g was applied along the z-axis, the operating frequency range was 6600–6700 Hz with a step of 2 Hz. All four structures were compressed, and the nanowires of all structures produce the same voltage [25], and the highest output voltage is observed at 6648 Hz. Fig 8(c) illustrates the relationship between frequency and voltage produced while applying an acceleration of 0.1 g along the z-axis. Fig 8(a), 8(b), and 8(c) highlight the accelerometer’s sensitivity within the specified frequency ranges of the x, y, and z axes, respectively.

**Fig 9:**
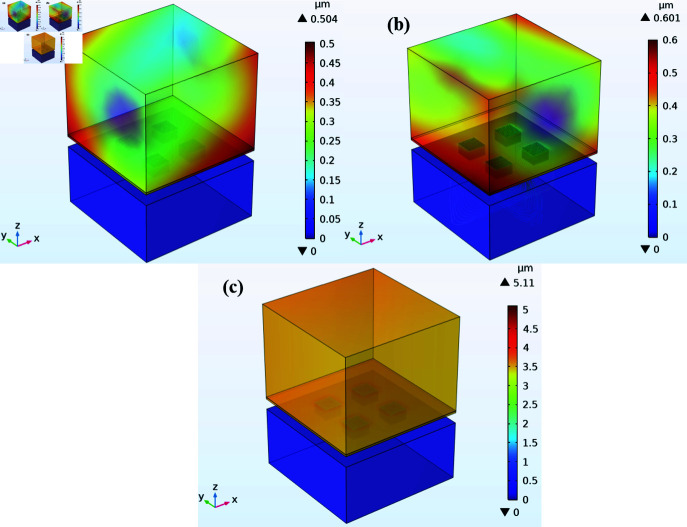
Deformation of accelerometer subjected to acceleration of 0.1 g applied in the direction of (a) X-Axis (b) Y-Axis (c) Z-Axis.

**Fig 10:**
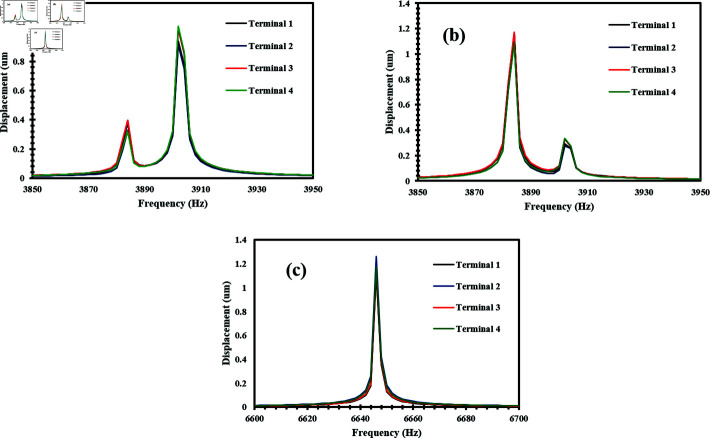
Frequency vs displacement graph of ZnO nanowires when an acceleration of 0.1 g is applied in the direction of (a) X-Axis (b) Y-Axis (c) Z-Axis.

Similarly, the deformation behavior of the nanowires was observed while applying a dynamic acceleration of 0.1 g across all three axes. Fig 9(a), 9(b), and 9(c) present the deformation of the accelerometer when acceleration was applied along the x-axis, y-axis, and z-axis. Fig 10(a), 10(b), and 10(c) illustrate the results of frequency vs deformation when acceleration was applied along the x-axis, y-axis, and z-axis respectively. The maximum deformation in the x-axis is reported at 3902 Hz, in the y-axis at 3882 Hz, and at 6648 Hz when acceleration was applied along the z-axis.

**Fig 11:**
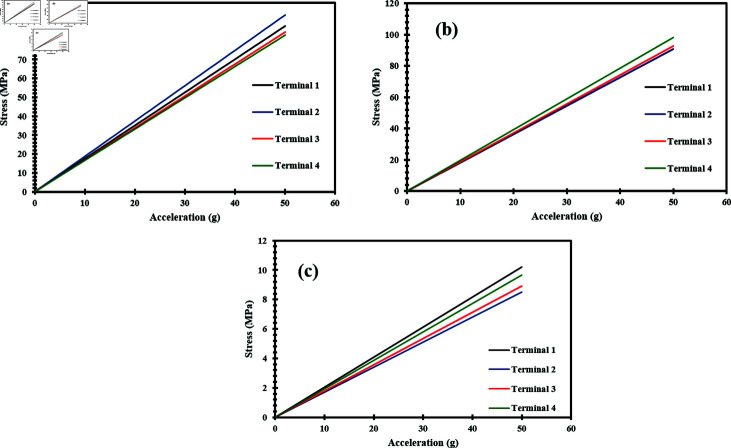
Acceleration vs stress graph of ZnO nanowires when a static acceleration up to 50 g is applied in the direction of (a) X-Axis (b) Y-Axis (c) Z-Axis.

In addition to dynamic acceleration, static acceleration was applied to the accelerometer across all three axes, ranging up to 50 g with a step of 1 g. Fig 11(a), 11(b), and 11(c) shows the relationship between applied acceleration and resulting stress when applied along the x-axis, y-axis, and z-axis respectively. The resulting stress demonstrates the linear relationship between acceleration and stress. This linear relationship illustrates the predictable and stable response of the ZnO nanowires under static loading conditions, which is crucial for ensuring the sensor’s reliability in various applications. The sensitivity of the accelerometer was reported under static acceleration as 1.8 MPa/g along the x-axis and y-axis, and 0.21 MPa/g along the z-axis.

**Fig 12:**
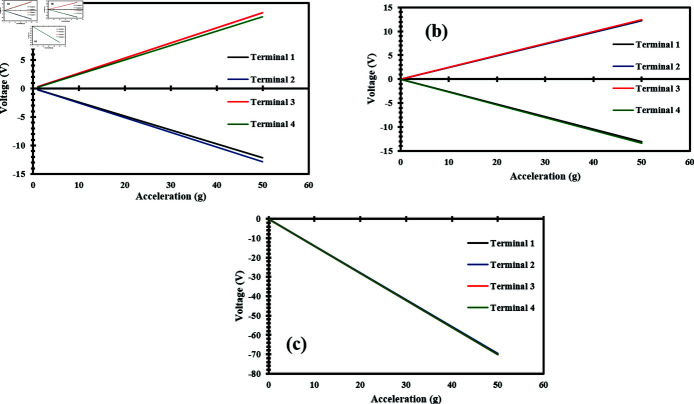
Applied acceleration vs generated voltage of ZnO nanowires when a static acceleration up to 50 g is applied in the direction of (a) X-Axis (b) Y-Axis (c) Z-Axis.

Similarly, static acceleration analysis was performed to analyze the generated voltage when subjected to acceleration along all three axes. The applied acceleration analysis range and step size were the same as in the case of acceleration vs stress analysis. Firstly, an acceleration is applied up to 50 g along the x-axis. Structures 1 and 2 experienced compressive forces, and due to the compression of the nanowires, they produced a negative voltage, while structures 3 and 4 experienced tensile forces, causing the nanowires to produce a positive voltage [28, 33]. Notably, structures 1 and 2 produced the same voltage magnitude as structures 3 and 4, but with opposite polarity. Fig 12(a) illustrates the output voltage produced when the acceleration was applied along the x-axis. Likewise, an acceleration was applied in the y-axis up to 50 g. In contrast, structures 1 and 4 experienced compression, causing the nanowires to produce a negative voltage, whereas structures 2 and 3 generated a positive voltage because they were subjected to tensile forces [28, 33].

Fig 12(b) shows the relationship between acceleration and output voltage when the acceleration is applied along the y-axis. Similarly, acceleration was applied along the z-axis up to 50 g. In this case, all the structures i.e. 1, 2, 3, and 4 experienced compressive stress. Due to the compressive stress, all structures generated an identical negative voltage [S3 dataset] [25]. Fig 12(c) presents the graph showing the relationship between the applied acceleration and generated voltage when it was applied along the z-axis. The results of acceleration and voltage demonstrate a linear relationship, with the voltage changing directly proportional to the applied acceleration. The analysis also revealed a sensitivity of 0.25 mV/g in the shear axis i.e. the x and y-axes, while 1.40 mV/g was observed in the normal axis i.e. the z-axis. The high value of sensitivity indicates the device’s high responsiveness to changes in acceleration. This underscores the robustness and reliability of the accelerometer design.

**Fig 13:**
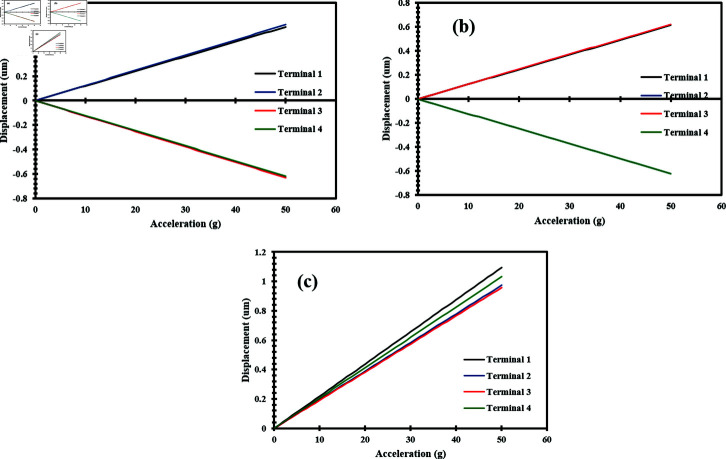
Applied acceleration vs deformation of ZnO nanowires when a static acceleration up to 50 g is applied in the direction of (a) X-Axis (b) Y-Axis (c) Z-Axis.

Static acceleration analysis was further extended to analyze the deformation of nanowires across all three axes. The applied acceleration range in this case was also up to 50 g with a step of 1 g. Fig 13(a), 13(b) and 13(c) depict the relationship between the applied acceleration and the deformation of nanowires when applied along the x-axis, y-axis, and z-axis respectively. The graphs illustrate that the deformation of the nanowires increases proportionally with the applied acceleration. The consistent linear increase across the x-axis, y-axis, and z-axis indicates the uniform mechanical response of the nanowires under static acceleration [S1 and S2 Datasets]. The sensitivity of the accelerometer was measured as 12 nm/g along the x-axis and y-axis, and 21 nm/g along the z-axis. The accelerometer’s sensitivity suggests that the sensor can detect minimal deformations when an acceleration is applied.

Piezoelectric accelerometers based on nanowires, as reported in the literature, are limited to measuring acceleration along a single axis, primarily in the normal direction. To the best of our knowledge, no prior studies have reported the development of an accelerometer that can measure acceleration in the shear axis by using nanowires, specifically along the x & y axis. Table 1 presents a comparative analysis of the proposed piezoelectric nanowire accelerometer with previous designs reported in the literature. The previously reported nanowires-based accelerometers mentioned in Table 1 can measure acceleration in only single axis, whereas the proposed accelerometer can measure acceleration in three axes. Moreover, the proposed sensor offers higher sensitivity and a wider measurement range. The proposed ZnO nanowires-based piezoelectric accelerometer can be used in biomedical applications to assist surgeons in surgeries [36–38]. They can also be utilized in various healthcare applications [24].

**Table 1:** Comparative analysis of previous studies with proposed sensors having piezoelectric nanowires.

Reference	Axis	Material	Thickness	Range	Sensitivity
Kim et al. [3]	Single Axis	ZnO	8 µm	0–1 g	15.1 pA/g
Koka et al. [20]	Single Axis	BaTiO_3_	45 µm	–	50 mV/g
Wang et al. [18]	Single Axis	ZnO	–	–	16.3 mV/g
Ramanay et al. [31]	Single Axis	ZnO	1.3 µm	0–1 g	6.9 V/g
Ramanay et al. [30]	Single Axis	ZnO	–	0–1 g	1.93 V/g
Song et al. [27]	Single Axis	ZnO	12 µm	0–1 g	37.7 pA/g
Proposed Sensor	Tri-Axis	ZnO	40 µm	0–50 g	Shear: 0.25 V/g
					Normal: 1.40 V/g

## Comparison of mathematical model and FEM simulations

To validate the results of FEM simulations, their results were compared with the mathematical model results. While applying static acceleration along the z-axis up to 50 g, the mathematical model predicted a sensitivity of 1.59 V/g, whereas the FEM simulations indicated a sensitivity of 1.40 V/g, which is slightly lower than the mathematical model results.

**Fig 14:**
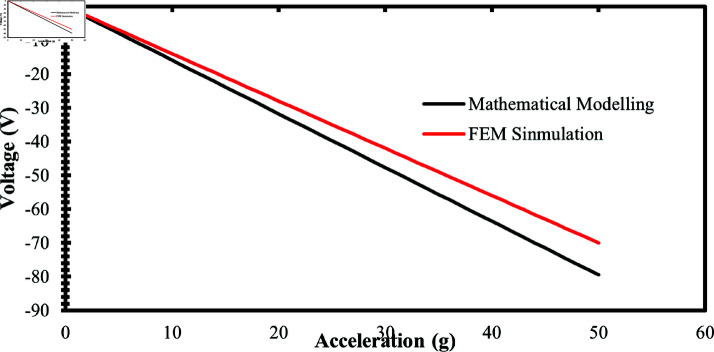
Comparison between mathematical modelling and FEM simulation.

Fig 14 illustrates the comparison between the mathematical model results and the FEM simulation results. The comparison graphs highlight the linear behavior of the accelerometer. The slight difference in sensitivity between the results is due to some idealized assumptions in the analytical model and FEM simulations, as well as additional factors such as boundary conditions.

The percentage difference between the results of the mathematical model and the FEM simulations was calculated using Eq 9.


$$\begin{array}{c} \%age\;Diff.=\frac{MathematicalModelSensitivity-FEMSimulationSensitivity}{MathematicalModelSensitivity}\times 100 \end{array}$$ (9)

The percentage difference between the mathematical model sensitivity and the FEM simulation sensitivity is 11.95%.

## Conclusion

This study has presented a comprehensive design and analysis of a ZnO nanowires-based piezoelectric accelerometer, emphasizing its capability to measure acceleration in three axes. An analytical model of the accelerometer was derived, and the FEM simulations were performed to analyze the behavior of the piezoelectric accelerometer. The accelerometer was tested by applying both static and dynamic acceleration. The FEM simulations revealed the resonance frequencies of the accelerometer to be 3902 Hz, 3883 Hz, and 6647 Hz along the x-axis, y-axis, and z-axis respectively. The accelerometer shows the linear output and high sensitivity under applied static acceleration. The sensor demonstrated a sensitivity of 0.25 V/g for in-plane acceleration and 1.40 V/g for out-of-plane acceleration. The unique properties of ZnO nanowires enhance the sensor’s sensitivity and enable it to function as a self-powered device. Its linear response under static acceleration makes it suitable for integration into biomedical applications.

## Supporting information

S1 Data Acquired dataset between acceleration and displacement of nanowire structures in x-direction. (XLXS)

S2 Data Acquired dataset between acceleration and displacement of nanowire structures in y-direction. (XLXS)

S3 Data Acquired dataset between acceleration and voltage of nanowire structures in z-direction. (XLXS)
